# Model thrombi formed under flow reveal the role of factor XIII-mediated cross-linking in resistance to fibrinolysis

**DOI:** 10.1111/j.1538-7836.2010.03963.x

**Published:** 2010-09

**Authors:** N J Mutch, J S Koikkalainen, S R Fraser, K M Duthie, M Griffin, J Mitchell, H G Watson, N A Booth

**Affiliations:** * Institute of Medical Sciences, University of AberdeenAberdeen; † University of LeedsLeeds; ‡ Aston UniversityBirmingham; § Aberdeen Royal InfirmaryAberdeen, UK

**Keywords:** factor XIII, fibrinolysis, flow, stability, thrombi.

## Abstract

**Summary:**

*Background:* Activated factor XIII (FXIIIa), a transglutaminase, introduces fibrin–fibrin and fibrin–inhibitor cross-links, resulting in more mechanically stable clots. The impact of cross-linking on resistance to fibrinolysis has proved challenging to evaluate quantitatively. *Methods:*We used a whole blood model thrombus system to characterize the role of cross-linking in resistance to fibrinolytic degradation. Model thrombi, which mimic arterial thrombi formed *in vivo*, were prepared with incorporated fluorescently labeled fibrinogen, in order to allow quantification of fibrinolysis as released fluorescence units per minute. *Results:*A site-specific inhibitor of transglutaminases, added to blood from normal donors, yielded model thrombi that lysed more easily, either spontaneously or by plasminogen activators. This was observed both in the cell/platelet-rich head and fibrin-rich tail. Model thrombi from an FXIII-deficient patient lysed more quickly than normal thrombi; replacement therapy with FXIII concentrate normalized lysis. *In vitro* addition of purified FXIII to the patient's preprophylaxis blood, but not to normal control blood, resulted in more stable thrombi, indicating no further efficacy of supraphysiologic FXIII. However, addition of tissue transglutaminase, which is synthesized by endothelial cells, generated thrombi that were more resistant to fibrinolysis; this may stabilize mural thrombi *in vivo*. *Conclusions:*Model thrombi formed under flow, even those prepared as plasma ‘thrombi’, reveal the effect of FXIII on fibrinolysis. Although very low levels of FXIII are known to produce mechanical clot stability, and to achieve γ-dimerization, they appear to be suboptimal in conferring full resistance to fibrinolysis.

## Introduction

Activation by thrombin of the transglutaminase (TG) factor XIII (FXIII) introduces cross-links into the fibrin matrix, dramatically altering its rheologic properties. The role of FXIII *in vivo* is clear, deficiency resulting in bleeding, usually after a delay, impaired wound healing and spontaneous abortion [1], a phenotype echoed in FXIIIA-deficient mice [2]. The human recessive autosomal condition usually arises from mutations in the A-subunit of FXIII [3], and is characterized in the laboratory by soft plasma clots that are soluble in urea and less mechanically stable [4]. Fibrin is a cofactor in FXIII activation, forming a ternary complex with thrombin [5] and facilitating release of the activation peptide and dissociation of the carrier B-subunit [4] to form the active enzyme, FXIIIa. In fibrin, the initial reaction catalyzed by FXIIIa is between Gln389/399 on one γ-chain and Lys406 on another, generating a γ–γ-dimer [6,7]. This is followed by generation of high molecular mass polymers of the α-chain [7], with multimeric cross-linked products of the γ-chain occurring over extended periods [8]. Another enzyme in the family, tissue TG (TG2) occurs in erythrocytes and endothelial cells [9]. TG2 exhibits a broader specificity than FXIIIa, catalyzing cross-linking between γ-chains and α-chains, and forming α-multimers in both fibrinogen and fibrin [10].

FXIIIa contributes to clot stability by cross-linking inhibitors of fibrinolysis, primarily α_2_-antiplasmin (α_2_AP), to fibrin, decreasing the susceptibility of clots to lysis [11]. Plasminogen activator inhibitor (PAI)-2 [12] and thrombin-activatable fibrinolysis inhibitor (TAFI) [13] are substrates for TGs, and can thus be incorporated into fibrin. Despite this body of evidence on cross-linked inhibitors, especially α_2_AP [14], there has been variability in visualizing the effect of FXIII in fibrinolytic assays, with several studies showing little, if any, effect [15–18], and others showing less efficient lysis of cross-linked clots [8,19–22]. Different explanations have been given for these discrepancies [8,15], but there is a need for a quantitative method that reveals the effect of cross-linking on fibrinolysis. Whole blood model thrombi formed under flow show a similar structure and protein distribution to thrombi formed *in vivo* [23], and have revealed the complementary nature of α_2_AP, PAI-1 and TAFI [24]. Here, we used model thrombi, and show that fibrinolysis is dramatically increased in FXIII deficiency, an effect that could be recapitulated by incorporating a non-reversible inhibitor of TGs.

## Materials and methods

### Blood collection and preparation of plasma

Peripheral blood was collected from consenting normal healthy donors into a 0.1 volume of 0.13 m trisodium citrate; for some experiments, platelet-free plasma was prepared [25] as a pool from 15 normal individuals (pooled normal plasma). Blood was also donated by a congenital homozygous FXIII-deficient patient (patient 1 in Anwar *et al.* [26]), characterized as having truncated FXIIIA, the result of mutations within the splice-donor sites. The patient was receiving routine prophylaxis with approximately 10 U kg^−1^ Fibrogammin® P (Aventis, Paris, France) at 4-weekly intervals, and blood samples were taken before this treatment unless otherwise stated.

### Thrombus formation and lysis

Thrombi were formed essentially as previously described [27,28]. Briefly, fluorescein isothiocyanate (FITC)-labeled fibrinogen (75 μg mL^−1^ final concentration; FITC/ fibrinogen approximately 6 : 1) was added to citrated whole blood (0.9 mL), and the system was recalcified by addition of 10.9 mm CaCl_2_ in a total volume of 1.15 mL. A non-reversible TG inhibitor, 1,3-dimethyl-2-[(2-oxopropyl) thio]imidazolium chloride (1 mm) [29], FXIII (1 or 2.5 U mL^−1^; Fibrogammin P) or guinea pig TG2 (1, 2 or 4 U mL^−1^; Sigma-Aldrich, Poole, UK) was added to blood prior to thrombus formation. The same method was used to prepare ‘thrombi’ from platelet-free plasma. After rotation at a constant speed of 30 r.p.m. for 90 min at room temperature, thrombi were removed from the serum and washed in 0.9% (w/v) NaCl. Thrombi were then bathed in 10 mm Tris (pH 7.5) and 0.01% Tween-20 containing tissue-type plasminogen activator (t-PA) at 1 μg mL^−1^ unless otherwise stated. In some experiments, thrombi were incubated in buffer alone, to examine spontaneous lysis, or with 1 μg mL^−1^ urokinase-type plasminogen activator (u-PA). Thrombi were incubated at 37 °C, samples of the supernatant (5 μL) were removed at 0 min and at 30-min intervals and diluted 1 : 50 in 10 mm phosphate and 150 mm NaCl (pH 7.4), and the fluorescence was then measured (excitation 485 nm; emission 530 nm). In some experiments, thrombi were bisected into cell-rich head and fibrin-rich tail, and lysed separately. Incorporation of FITC–fibrinogen was analyzed by lysing heads and tails to completion (18 h at 37 °C in 1 μg mL^−1^ t-PA and 100 μg mL^−1^ plasminogen).

### FXIII activity assay

TG activity in plasma was quantified by using an adaptation of two methods [30,31]. Human fibronectin (5 μg per well) was used to coat 96-well plates (CoStar; Corning, Lowell, MA, USA). The FXIII standard was pooled normal plasma, standardized against the international standard [32], preactivated with 1 U mL^−1^ bovine thrombin at 37 °C for 5 min. Residual thrombin was neutralized by hirudin (2 μg mL^−1^), and samples were diluted in 0.1 m Tris (pH 7.4) and 1 mm dithiothreitol (DTT), to construct a standard curve. Guinea pig tissue TG (Sigma) activity was measured in the same way but without prior thrombin treatment. The TG reaction, in 0.1 m Tris (pH 7.4), 1 mm DTT, 5 mm CaCl_2_ and 0.5 mm 5-(biotinamido)pentylamine (Pierce Thermo Fisher Scientific, Rockford, IL, USA ), was stopped after 2 h at 37 °C by addition of 2 mm EDTA in 0.1 m Tris (pH 7.4). Plates were washed and blocked with 0.5% (w/v) milk powder for 30 min at 37 °C, and incorporated biotinylated amine was detected [30]. The assay was linear for plasma FXIIIa between 2.5% and 100% normal, prepared by mixing FXIII-deficient plasma (Affinity Biologicals, Ancaster, Canada) and pooled normal plasma. The coefficient of variation was 7%, based on nine independent assays of 50% normal plasma.

### Sodium dodecylsulfate polyacrylamide gel electrophoresis (SDS-PAGE)

Cross-linked fibrin was analyzed by clotting 10% (v/v) plasma samples with final concentrations of 15 mm cysteine, 8 mm CaCl_2_ and 0.2 U mL^−1^ thrombin in glass tubes [33]. Clots were harvested after 30 min at 37 °C by winding onto thin glass rods (1.5 mm diameter), washed with 10 mm EDTA in 0.9% (w/v) NaCl, dried in air, and dissolved in reducing buffer (10 min at 72 °C, before separation on 4–12% polyacrylamide Bis–Tris NuPAGE gels; Invitrogen, Karlsruhe, Germany). Degradation products from plasma ‘model thrombi’, after 4 h of lysis, were analyzed under non-reducing conditions on the same gels. Gels were stained with Coomassie Blue Brilliant R or immunoblotted with antibody to the fibrinogen γ-chain (Santa Cruz Biotechnology, Santa Cruz, CA, USA).

### Data analysis

Quantitative data are expressed as the mean and standard error of the mean (*n* = at least 3). Data were analyzed in GraphPad Prism 5 (GraphPad Software, La Jolla, CA, USA) and shown as fluorescence units (FU) released; rates of lysis (FU min^−1^) were determined by linear regression, and used to calculate fold differences. Statistical analysis was performed by *t*-test, and *P*-values < 0.05 were considered to be significant.

## Results

### TGs stabilize model thrombi

**Fig. 1:**
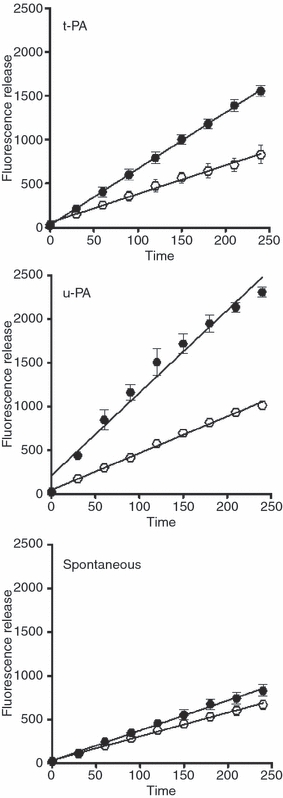
Inhibition of cross-linking in thrombi. Thrombi were prepared in the absence (○) or presence (•) of transglutaminase (TG) inhibitor and bathed in fluid containing tissue-type plasminogen activator (t-PA; 1 μg mL^−1^, *n* = 5), urokinase-type plasminogen activator (u-PA; 1 μg mL^−1^, *n* = 4) or buffer alone (spontaneous, *n* = 8). Lysis was monitored as release of fluorescence and expressed as mean ± standard error of the mean. Differences in lysis rate with addition of TG inhibitor were significant (*P* < 0.01) in all cases.

Model thrombi were formed in the presence and absence of a non-reversible TG inhibitor [29]. The inhibitor was used at 1 mm, which is more than a 100-fold higher than its inhibition constant, based both on the original work [29] and on our analysis of its efficiency in inhibiting plasma FXIIIa activity, where the IC_50_ was found to be 7 μm (data not shown). Incorporation of TG inhibitor into the forming model thrombus doubled the rates of lysis, relative to no inhibitor. This was evident for lysis induced by t-PA (Fig. 1; 2.0-fold increase in rate of lysis; *P* < 0.005) or u-PA (Fig. 1; 2.2-fold increase; *P* < 0.001), present at 1 μg mL^−1^ in the bathing fluid surrounding the washed thrombus. Model thrombi have previously been shown to lyse spontaneously in the absence of added plasminogen activators [34]. Inhibition of cross-linking had less impact on spontaneous lysis (Fig. 1), but TG inhibitor still increased the lysis rate significantly (1.2-fold increase; *P* < 0.005).

**Fig. 2:**
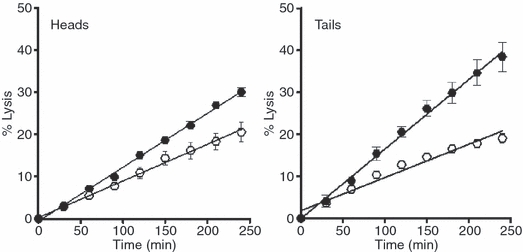
Cross-linking in heads and tails of thrombi. Thrombi were prepared in the absence (○) or presence (•) of transglutaminase (TG) inhibitor. The thrombi were bisected into the platelet-rich head and fibrin-rich tail before being lysed separately with tissue-type plasminogen activator (t-PA; 1 μg mL^−1^). Lysis was monitored as release of fluorescence, and results are expressed as mean lysis (%) ± standard error of the mean (*n* = 4) relative to values obtained when thrombi were lysed to completion with additional plasminogen and t-PA, as described in Materials and methods. Differences in percentage lysis with addition of TG inhibitor were significant (*P* < 0.005) for both heads and tails.

Model thrombi have a defined structure, with a cell-rich and platelet-rich head, and a fibrin-rich tail [23]. We separately lysed the heads and tails of model thrombi with t-PA (Fig. 2) or u-PA (data not shown). These data are presented as percentage lysis, because incorporation of FITC–fibrinogen was found to be consistently higher (about 1.5-fold) in heads than in tails, as assessed by lysis to completion with t-PA and added plasminogen. TG inhibitor increased lysis of both heads (1.5-fold increase) and tails (2.1-fold increase), both increases being significant (*P* < 0.005). Thus, the effect of cross-linking was clear throughout the thrombus.

### Stability of FXIII-deficient thrombi

**Fig. 3:**
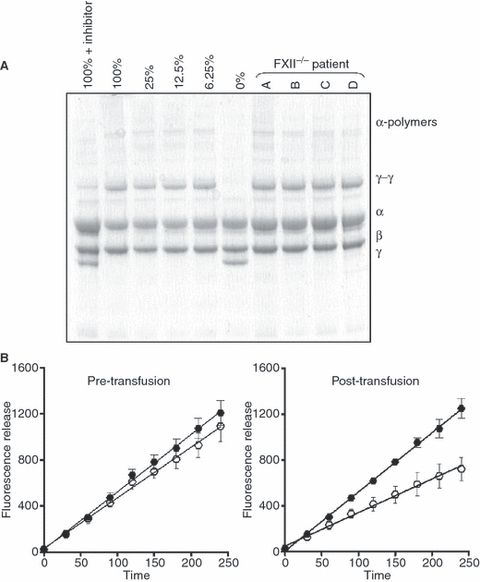
The role of plasma FXIII in plasma clots and thrombus stabilization. (A) Plasma clots were formed with pooled normal plasma (PNP), or mixtures of PNP with FXIII-deficient plasma to give different concentrations of FXIII relative to normal (1.5–100%). Clots were also prepared from the plasma of an FXIII-deficient patient that was collected pre-transfusion on four separate occasions (A–D). After 30 min, clots were harvested and subjected to sodium dodecylsulfate polyacrylamide gel electrophoresis before staining for protein with Coomassie Brilliant Blue R. (B) Blood was collected from the patient pre-transfusion and post-transfusion with FXIII (Fibrogammin P) on four separate occasions [as in (A)]. Thrombi were formed in the absence (open symbols) or presence (closed symbols) of transglutaminase (TG) inhibitor, and lysed with tissue-type plasminogen activator (1 μg mL^−1^). Lysis was monitored as release of fluorescence, and results are expressed as mean ± standard error of the mean (*n* = 4). Differences in lysis rate with addition of TG inhibitor were significant (*P* < 0.005) both pre-treatment and post-treatment.

Blood samples were collected from a congenitally homozygous FXIII-deficient patient before and after routine FXIII administration. His preadministration plasma TG activity, after activation with thrombin, was 8.8% ± 2.8% [mean ± standard deviation (SD)] of normal. Clots prepared from the patient plasma and analyzed by SDS-PAGE were close to normal in terms of γ–γ-dimer detection (Fig. 3A). Fully deficient commercial plasma or mixtures with < 3% normal FXIII showed detectable γ-monomer, as did normal plasma treated with TG inhibitor.

**Fig. 4:**
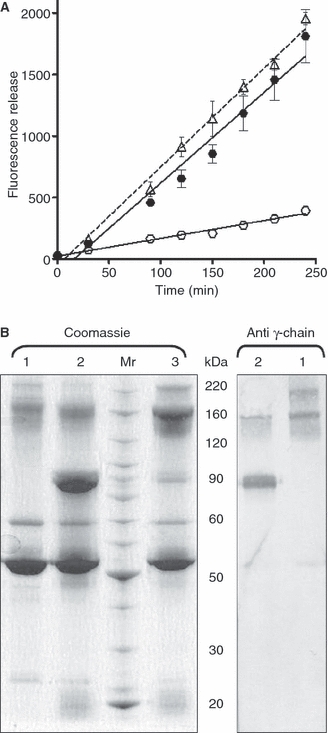
Cross-linking stabilizes plasma ‘thrombi’ and alters the pattern of fibrin degradation products. (A) Plasma from normal individuals was used to form ‘thrombi’, in the absence (○) and the presence (•) of transglutaminase (TG) inhibitor, and these were compared with plasma ‘thrombi’ from the FXIII-deficient patient (Δ). Lysis with tissue-type plasminogen activator (1 μg mL^−1^) was recorded as release of fluorescence over time, and results are expressed as mean ± standard error of the mean (*n* = 3). The difference in lysis rate upon addition of TG inhibitor to plasma ‘thrombi’ was significant (*P* < 0.005), as was the difference between the normal control and the patient (*P* < 0.005). (B) After the final time point (4 h), samples were taken from the bathing fluid of the model thrombi formed in the absence (1) and presence (2) of TG inhibitor or from the FXIII-deficient patient (3). Samples were separated on 4–12% polyacrylamide gels before staining in Coomassie Brilliant Blue R or transferring to nitrocellulose and staining with an antibody to the γ-chain of fibrinogen.

Model thrombi prepared from the patient's blood were lysed more quickly by t-PA (4.4 ± 0.5 FU min^−1^; *n* = 4) than those from normal controls (2.7 ± 0.1 FU min^−1^; *n* = 19; *P* < 0.005). The effect of TG inhibitor on patient thrombi was small, but still significant (Fig. 3B; 1.1-fold increase; *P* < 0.005). Thirty minutes after routine 4-weekly prophylaxis with Fibrogammin (10 U kg^−1^), plasma activity rose to 54.9% ± 7.9% of normal. The thrombi from post-FXIII administration blood had normal rates of lysis (2.9 ± 0.1 FU min^−1^ vs. 2.7 ± 0.1 FU min^−1^, respectively; *P* = 0.08), and the greater effect of TG inhibition was restored (Fig. 3; 1.8-fold increase; *P* < 0.005). Similar results were achieved when different concentrations of t-PA and u-PA (0.25–1 μg mL^−1^) were used for lysis or for spontaneous lysis in the absence of plasminogen activator (not shown). We then formed ‘thrombi’ from platelet-free plasma. Their lysis revealed a 5.1-fold increase in lysis rate (Fig. 4A; *P* < 0.005) and a significant contrast between normal and FXIII-deficient plasma (5.6-fold increase; *P* < 0.005). Bathing fluid harvested after the final time point (4 h) was analyzed by SDS-PAGE and western blot. A change in the pattern of fibrin degradation products was observed, especially in terms of accumulation of the D-monomer (90 kDa) band, which was present when the TG inhibitor was added to normal plasma. This band was also apparent, but at a lesser intensity, in bathing fluid from the patient plasma ‘model thrombi’ (Fig. 4B).

### Further stabilization of thrombi by addition of FXIII or tissue TG

**Fig. 5:**
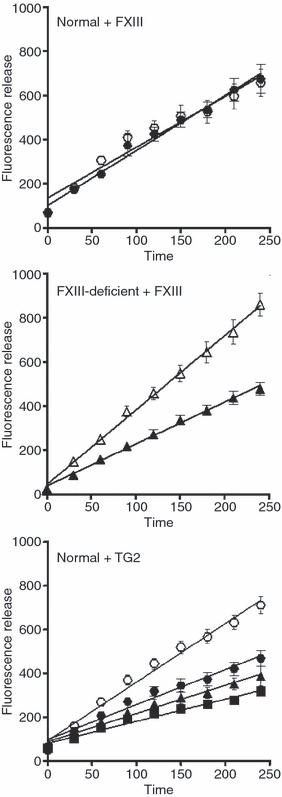
Additional stabilization of normal and FXIII-deficient blood. Thrombi were formed from the whole blood of normal subjects or from the patient deficient in FXIII with the addition of FXIII or transglutaminase 2 (TG2). Thrombi were lysed with tissue-type plasminogen activator (1 μg mL^−1^), and lysis was recorded as release of fluorescence. The normal control data in the top and bottom panels are mean value ± standard error of the mean of 19 different subjects. The difference in lysis with addition of FXIII (1 U mL^−1^ Fibrogammin P; •, ▴) was not significant in normal subjects (*P* = 0.15, *n* = 3) but was significant in thrombi from FXIII-deficient blood (*P* < 0.001, *n* = 3). Addition of 1 U mL^−1^ (•), 2 U mL^−1^ (▴) and 4 U mL^−1^ (▪) TG2 (*n* = 3) resulted in significantly less lysis (*P* < 0.005) relative to the normal control and each concentration tested.

Addition of Fibrogammin to normal blood had no effect on the stability of model thrombi (Fig. 5; *P* = 0.15), but addition to FXIII-deficient blood consistently made the thrombi more resistant to lysis (Fig. 5; *P* < 0.001; 1.8-fold decrease). For clarity, only the addition of 1 U mL^−1^ Fibrogammin to normal blood is shown, but similar results were obtained with 2.5 U mL^−1^. The data show that supraphysiologic levels of FXIII produce no more resistance to lysis than normal levels. Fibrin can also be cross-linked by TG2 with a pattern that is distinct from that with FXIII [10,35]. To determine whether TG2 could confer an additional degree of resistance to fibrinolysis, we added TG2 to normal blood (Fig. 5) at concentrations equivalent to that of Fibrogammin, on the basis of activity assay values. Such additions of TG2 caused clear dose-dependent decreases in lysis, with reductions of 1.7-fold, 2.1-fold and 2.6-fold for 1, 2 and 4 U mL^−1^, respectively. These differences were significant (*P* < 0.005) relative to no addition and for each concentration relative to the others. These data show that TG2 stabilization of thrombi against fibrinolytic degradation supplements that caused by FXIIIa.

## Discussion

A causal relationship between FXIII-dependent cross-linking and decreased fibrinolysis is predicted and often assumed, but its quantitative demonstration in the laboratory has been controversial [8,15–22]. This study reports the use of a flow model [23,24,28] to examine model thrombus lysis. Thrombi were prepared with blood from an FXIII-deficient patient, which lysed much faster than thrombi from normal blood. Similarly enhanced lysis was achieved by inhibiting cross-linking in normal blood. We used a TG inhibitor that alkylates the active site cysteine of the TGs without influencing other thiol-sensitive enzymes [29], and is thus a more selective tool than those that react with all cysteine groups, such as cystamine or iodoacetamide, or chelate calcium ions [36]. Inclusion of TG inhibitor during whole blood thrombus formation under flow, with shear rates equivalent to those found in larger arteries (400–600 s^−1^) [28], reproducibly doubled the rate of fibrinolysis. The effect of TG inhibition was apparent in both heads and tails of model thrombi, suggesting the importance of cross-linking throughout the entire thrombus.

The data presented here demonstrate an effect of inhibiting cross-linking on fibrinolysis, whether t-PA or u-PA was used to activate plasminogen, and also on the spontaneous lysis of model thrombi, owing primarily to u-PA activity from polymorphonuclear cells [34]. These cells have been shown to degrade FXIII [37], but, clearly, sufficient native FXIII remains in model thrombi to achieve cross-linking. The concentration of t-PA or u-PA (1 μg mL^−1^) was chosen to reflect pharmacologic conditions and to avoid very high concentrations, which result in inefficient lysis [28], but the effects of neutralizing TG activity were also apparent at 250 ng mL^−1^ t-PA and when no activator was added (not shown). Our data expand and quantify the observation made in 1966, in a similar flow system, with lysis assessed simply by inspection [19]. Formation of thrombi under flow has emerged from our studies as an essential feature in revealing sensitivity to cross-linking. Parallel studies using static clots, prepared with incorporated FITC–fibrinogen, have failed to show reproducible effects of cross-linking; the same was true of turbidity assays [12,14,27] (not shown). The observation that even plasma ‘model thrombi’, formed under flow, demonstrate sensitivity to cross-linking greatly increases the utility and convenience of the model system, allowing the use of stored plasma.

The patient used in this study (patient 1 in [26]) is well controlled by routine monthly prophylaxis with FXIII concentrate. He has had no spontaneous bleeds during the last 9 years. At the typical nadir of 8–9% normal plasma FXIII, γ–γ-dimers were apparent in fibrin prepared from his plasma. Plasma clots from the same patient, some 25 years ago, when he was treated less regularly and with fresh frozen plasma rather than FXIII concentrate, had barely detectable γ–γ-dimers [33]. Our model thrombus system shows clearly that 8–9% normal plasma FXIII is not sufficient to achieve normal thrombus resistance to lysis, whereas the literature suggests that trace levels of FXIII are adequate for normal hemostasis [1,3,4]. Even at 8-9% FXIII activity, when stability of thrombi was severely compromised, it was possible to detect the effect of inhibiting TG, highlighting the exquisite sensitivity of our system to cross-linking. Definitions of normality are inevitably dependent on the methods used. The established view, that as little as 5% FXIII is sufficient, comes primarily from studies on clotting [38]. Normality in terms of fibrinolysis is not routinely assessed, but Figure 1C shows clearly that spontaneous lysis is sensitive to cross-linking. The challenge of exposing model thrombi to pharmacologic concentrations of t-PA reveals a requirement for higher levels of FXIII in this setting. The data on the patient after administration of FXIII show that plasma samples containing about 50% normal FXIII are indistinguishable from normal plasma in terms of model thrombus stability. More detailed studies will be necessary to define absolute requirements for FXIII in relation to different physiological and therapeutic circumstances, and such additional information will be useful in the context of FXIII being used during surgery in patients with propensity for bleeding [39,40].

The role of FXIII in enhancing the clot strength and elasticity of fibrin has been described extensively in static clots [41–43]. A similar inhibitor to that used here has been shown to have a profound effect on clot rigidity [43]. Clot strength is attributed largely to cross-linking of fibrin α-chains, the role of γ-chain dimers being more controversial [22,43,44]. Effects on lysis are even more variable, some studies showing no or minor effects of cross-linking [15–18], and others showing a relationship between cross-linking and poorer lysis [8,19–22]. Assays of fibrinolysis are well known to show variable sensitivity to different components, depending on several factors, particularly the balance between plasminogen activators and inhibitors [45], but also reflecting fibrinogen concentration [46], concentration and access of plasminogen [47], and ionic strength [8]. It is not surprising, therefore, that there is disagreement in the literature on the effects of cross-linking on fibrinolysis *in vitro*, but the impact of TGs on fibrinolytic resistance has been visualized *in vivo* with the use of an experimental model of pulmonary embolism [48]. Lysis of purified fibrin was not affected by γ–γ-dimers [21], whereas it was slowed by multimeric cross-linking of α-chains [8] and the formation of γ-multimers over extended periods [49]. The products of lysis in this study showed a clear band of D-monomer from patient samples or when cross-linking in normal plasma was inhibited, but our analysis was limited to samples taken after 4 h of lysis. This study was not designed to distinguish between stabilization resulting from fibrin–fibrin cross-links and those resulting from fibrin–inhibitor cross-links. Fibrin to which α_2_AP is cross-linked lysed more slowly both *in vitro* [11,50], and *in vivo* [48]. PAI-1, α_2_AP and TAFI all contribute to the stability of cross-linked clots and thrombi [24]. The sensitivity of model thrombi to different regulators of fibrinolysis shows its potential for defining the contributions of fibrin–fibrin and fibrin–inhibitor cross-links to resistance to fibrinolysis. Such model thrombi may be useful in the study of α_2_AP deficiency, as this inhibitor is markedly affected by cross-linking status [11,50] and, indeed, occurs cross-linked to fibrinogen in plasma [51].

TG2 is known to catalyze cross-linking of fibrin, with some distinctions in the exact pattern as compared with FXIIIa [9,10,35]. For this reason, we added TG2 to normal blood to determine whether the flow system was sensitive to the effects of TGs other than FXIIIa. We found that supplementing normal blood with TG2 produced thrombi that were more resistant to lysis, demonstrating an additional degree of fibrinolytic resistance over that observed with endogenous FXIII. Erythrocytes trapped within the fibrin network have been proposed as a source of TG2 [4], but our data imply limited release during thrombus formation. *In vivo*, it is likely that TG2 is present in mural thrombi, as it is an abundant protein in the vessel wall [52] and can be upregulated by thrombin [30]. It is noteworthy that active FXIIIa has a half-life of 20 min *in vivo* and has been reported to be a feature of new thrombi [53] but constitutively active TG2 may stabilize mature thrombi.

In conclusion, model thrombi prepared under flow are sensitive to the impact of cross-linking on fibrinolytic resistance. The system provides a convenient model in which to address many remaining questions, such as role of cellular FXIII, the influence of fibrin vs. inhibitor cross-linking, and the ability of different TGs to regulate these processes.
